# OA07.03. Randomized, double-blind, double-dummy trial of myrrh, chamomile, coffee charcoal compared to mesalazine in maintaining remission in ulcerative colitis

**DOI:** 10.1186/1472-6882-12-S1-O27

**Published:** 2012-06-12

**Authors:** J Langhorst, A Westendorf, M Knopp, S Schneider, K Goos, U Albrecht, A Rueffer, R Stange, A Michalsen, G Dobos

**Affiliations:** 1 University of Duisburg, Complementary and Integrative Medicine, Essen, Germany; 2 Department for Microbiology, University of Duisburg-Essen, Essen, Germany; 3 Biometric Institute, University of Hannover, Hannover, Germany; 4 Repha GmbH, Hannover, Germany; 5 Mediconomics, Hannover, Germany; 6 Enterosan, L+S Labor, Bad Bocklet - Grossenbrach, Germany; 7 Immanuel Hospital, Charité, Berlin, Germany

## Purpose

We compared the efficacy of the herbal preparation of myrrh, chamomile extract and coffee charcoal (herb) with a mesalazine (mes) therapy in maintaining remission in ulcerative colitis (UC).

## Methods

A total of 96 patients (51 female) with UC in remission (not longer than 12 months) were included in a randomized, double-blind, double-dummy, multicenter, non inferiority study comparing mesalazine 500 mg (3x1/d) to 100mg myrrh, 70mg chamomile extract and 50mg coffee charcoal (3 x 4/d) over a time period of 12 months. As primary outcome criterion, non-inferiority of the herbal preparation was defined and accepted, if the difference in the colitis activity index (Colitis Activity Index - CAI - Rachmilewitz) (calculated at six time points during the 12 month interval) averaged over all visits was ≤ 1 point. Furthermore, relapse rates, relapse-free times, safety, a comprehensive activity index (CAI, CRP and fecal Lactoferrin, Calprotectin and PMN-Elastasis), an endoscopic activity index and Health-related Quality of life (HrQoL) were assessed. Peripheral CD4+CD25+ reg T-cells were investigated in a subgroup at each time point and during a flare.

## Results

Primary outcome criterion (p = 0.19), relapse rates (CAI>4) (mes 22/49 patients vs herb 25/47 patients; p = 0.54), relapse-free time (268 ± 22 days for mes and 240 ± 23 days (p = 0.40) for the herb), the comprehensive activity index and HrQoL did not show a significant difference. Of notice, peripheral CD4+CD25+ regulatory T-cells showed a distinct different pattern at time points pre-flare and flare for the two treatment modalities (CD4+CD25+T_reg_ mes p=non significant (ns); herb p=0.02; CD4+CD25+ T_reg_ high mes p=ns; herb p=0.008).

## Conclusion

The herbal preparation shows efficacy and safety in maintaining remission non-inferior to mesalazine in ulcerative colitis. It appears to offer an alternative option for maintenance therapy. Regulatory T-cell pattern might give first evidence to suggest a different mechanism of action.

